# The efficacy of suppressive antibiotic treatment in patients managed non-operatively for periprosthetic joint infection and a draining sinus

**DOI:** 10.5194/jbji-6-313-2021

**Published:** 2021-08-17

**Authors:** Karel-Jan Dag François Lensen, Rosa Escudero-Sanchez, Javier Cobo, Rihard Trebše, Camelia Gubavu, Sara Tedeschi, Jose M. Lomas, Cedric Arvieux, Dolors Rodriguez-Pardo, Massimo Fantoni, Maria Jose Garcia Pais, Francisco Jover, Mauro José Costa Salles, Ignacio Sancho, Marta Fernandez Sampedro, Alex Soriano, Marjan Wouthuyzen-Bakker, ESCMID Study Group of Implant Associated Infections (ESGIAI)

**Affiliations:** 1 Department of Medical Microbiology and Infection Prevention, University Medical Center Groningen, University of Groningen, Groningen, the Netherlands; 2 Department of Infectious Diseases, Hospital Ramón y Cajal, IRYCIS, Madrid, Spain; 3 Service for Bone Infections, Valdoltra Orthopaedic Hospital, Ankaran, Slovenia; 4 Department of Infectious and Tropical Diseases, CHR Orléans, Orléans, France; 5 Infectious Diseases Unit, Department of Medical and Surgical Sciences, University of Bologna Policlinico di Sant Orsola, Bologna, Italy; 6 Department of Infectious Diseases, Virgen del Rocio University Hospital, Seville, Spain; 7 Department of Infectious Diseases and Intensive Care, Centre Hospitalier Universitaire de Rennes, Rennes, France; 8 Infectious Diseases Department, Vall d'Hebron Hospital Universitari, Vall d'Hebron Barcelona Hospital Campus, Passeig Vall d'Hebron 119-129, 08035 Barcelona, Spain; 9 UOC Malattie Infettive, Fondazione Policlinico Universitario A. Gemelli IRCCS, Rome, Italy; 10 Infectious Diseases Unit, Hospital Universitario Lucus Augusti, Instituto de Investigación Sanitaria de Santiago de Compostela (IDIS), Lugo, Spain; 11 Unidad de Enfermedades Infecciosas, Hospital Universitario San Juan de Alicante, Alicante, Spain; 12 Unit of Infectious Diseases, Department of Internal Medicine, Santa Casa de Misericórdia de São Paulo, São Paulo, Brazil; 13 Department of Infectious Diseases, Hospital Reina Sofía de Tudela, Navarra, Spain; 14 Servicio de Enfermedades Infecciosas, Hospital Universitario Marqués de Valdecilla, Santander, Spain; 15 Department of Infectious Diseases, Hospital Clínic, IDIBAPS, Catalonia, Barcelona, Spain; ➕ A full list of authors appears at the end of the paper

## Abstract

**Objectives**: Patients with prosthetic joint infections (PJIs) not suitable for curative surgery may benefit from suppressive antibiotic therapy (SAT). However, the usefulness of SAT in cases with a draining sinus has never been investigated. **Methods**: A multicentre, retrospective observational cohort study was performed in which patients with a PJI and a sinus tract were eligible for inclusion if managed conservatively and if sufficient follow-up data were available (i.e. at least 2 years). SAT was defined as a period of > 6 months of oral antibiotic therapy. **Results**: SAT was initiated in 63 of 72 (87.5 %) included patients. Implant retention during follow-up was the same in patients receiving SAT vs. no SAT (79.4 % vs. 88.9 %; p=0.68). In total, 27 % of patients using SAT experienced side effects. In addition, the occurrence of prosthetic loosening in initially fixed implants, the need for surgical debridement, or the occurrence of bacteremia during follow-up could not be fully prevented with the use of SAT, which still occurred in 42 %, 6.3 %, and 3.2 % of cases, respectively. However, the sinus tract tended to close more often (42 % vs. 13 %; p=0.14), and a higher resolution of pain was observed (35 % vs. 14 %; p=0.22) in patients receiving SAT. **Conclusions**: SAT is not able to fully prevent complications in patients with a draining sinus. However, it may be beneficial in a subset of patients, particularly in those with pain or the hindrance of a draining sinus. A future prospective study, including a higher number of patients not receiving SAT, is needed.

## 1 Introduction

Patients with a periprosthetic joint infection (PJI) require surgery in order to cure the infection. This can either be done with surgical debridement, antibiotics, and implant retention (DAIR) in case of an acute infection with a fixed implant or by extraction of the prosthesis in case of a chronic infection (Osmon et al., 2013). However, in some cases, surgical intervention is not an attractive option, for example, in old and fragile patients with multiple comorbidities or due to technical challenges with a high risk of amputation. In addition, some patients may themselves refuse surgery. For these patients, suppressive antibiotic treatment (SAT) might be an alternative option to maintain infection control and to reduce the risk of complications (Tsukayama et al., 1991; Segreti et al., 1998; Prendki et al., 2014; Rao et al., 2003; Siqueira et al., 2015; Wouthuyzen-Bakker et al., 2017; Pradier et al., 2018; Escudero-Sanchez et al., 2020). It can be debated if patients with a draining sinus benefit from SAT, especially for those where the infection is caused by a low virulent pathogen, where patients have low inflammatory parameters, or in the absence of pain. Indeed, in a recent survey, 10 % of physicians prefer to withhold SAT in patients with a draining sinus, and 50 % indicate that they consider withholding SAT in a select patient category (Lensen et al., 2020). The clinical outcome of both strategies in patients with a sinus tract is unclear. For this reason, we conducted a multicentre observational study with the aim of achieving the following outcomes:

i. describing the clinical outcome of inoperable patients treated with SAT and a draining sinus
ii. investigating whether the above-mentioned patients treated with SAT have a comparable clinical outcome with patients for whom SAT was withheld.

## 2 Material and methods

### 2.1 Study design

We performed a multicentre retrospective observational cohort study. There were 15 participating medical centres from six different countries, including Spain (n=8), France (n = 2), Italy (n = 2), Brazil (n = 1), Slovenia (n = 1), and the Netherlands (n = 1).

### 2.2 Patient selection

Adult, i.e. 18 years or older, PJI patients with a sinus tract were eligible for inclusion when the sinus tract was diagnosed between January 2008 and January 2018 and when they were considered ineligible for a potential curative surgical strategy or the patients themselves refused surgery. Patients were excluded if the duration of follow-up was less than 2 years. Some of the patients included in this study were part of a previously published article on the outcomes of SAT (Escudero-Sanchez et al., 2020).

The primary end point of this study was retention of the implant during follow-up. Secondary end points consisted of the prevention of prosthetic loosening in initially fixed implants, the need for surgical debridement during follow-up, closing of the sinus tract, resolution of pain, the development of bacteremia, the resolution of inflammation and anaemia, and side effects when treated with SAT. For this study, SAT was defined as a period of > 6 months of oral antibiotic therapy.

### 2.3 Study procedures

Data were collected by an individual physician or researcher at each of the participating centres. Demographic characteristics included age, sex, body mass index (BMI), smoking status, relevant comorbidities (e.g. diabetes mellitus, chronic kidney disease, and liver cirrhosis), and the affected joint. The indication to prescribe or withhold SAT was noted, and the data on the primary and secondary outcome parameters were collected.

### 2.4 Statistical analysis

Continuous variables are summarised by the mean (standard deviation) or median (interquartile range), depending on the normality of the data. Categorical variables are presented as frequencies or percentages. To compare the outcomes between SAT and non-SAT patients, we used a t test for normally distributed and the Mann–Whitney U test for non-normally distributed continuous data, whereas Fisher's exact test was used to compare categorical variables. Logistic regression analysis was performed to establish predictors of prosthesis retention. Spearman's ρ was used to establish correlations between haemoglobin and C-reactive protein (CRP) levels. IBM SPSS version 23.0 was used for the statistical analysis.

## 3 Results

### 3.1 Patient population

Table 1 shows the patient and implant characteristics of the total cohort of 72 included patients. The mean age was 74 years (standard deviation, SD, 15), of which 61 % were male. The mean BMI was 29.0 kg/m${}^{2}$ (SD 8.0). Approximately 20 % of patients were treated for diabetes mellitus, 4 % had chronic kidney disease, and 3 % had liver cirrhosis. Most of the included joints were hips and knees (50 % and 44 %, respectively), whereas only a small minority of the patients had a PJI of the shoulder or elbow (both 3 %). SAT was initiated in 63 of 72 (87.5 %) patients, for the following reasons: (i) common practice in the participating hospital in 22 out of 63 (35 %) cases, (ii) the intention to stop the drainage or close the sinus tract in 6 out of 63 (9.5 %) cases, (iii) the intention to prevent bacteremia in 5 out of 63 (8 %) cases, or (iv) a combination of the previous reasons in 10 out of 63 (16 %) cases. In 20 out of 63 (31.7 %) cases, an alternative reason or no indication was specified. In almost half of all cases (47.6 %), patients were treated with intravenous antibiotics prior to the start of SAT. Reasons for not initiating SAT were not noted but, in general, were based on the experience of an acceptable outcome in patients with a draining sinus.

**Table 1:** Patient and implant characteristics.

Patient and implant characteristics	(n=72)
Mean age (SD)	74 (15)
Male/female	44/28 (61 %/39 %)
Mean BMI (SD)	29.0 (8.0)
Smoker	4 (5.6 %)
Diabetes mellitus	15 (20.8 %)
Chronic kidney disease	3 (4.2 %)
Liver cirrhosis	2 (2.8 %)
Joint – Hip – Knee – Shoulder – Elbow	36 (50 %) 32 (44.4 %) 2 (2.8 %) 2 (2.8 %)
Primary prosthesis	26 (36.1 %)
Cemented prosthesis	39 (54.2 %)
SAT	63 (87.5 %)
IV antibiotics prior to SAT	30/63 (47.6 %)
Follow-up duration in months (SD)	54.4 (27)
Time (months) between appearance sinus tract and initiating SAT (IQR)	2.0 (0.0–8.0)

BMI – body mass index; SAT – suppressive antibiotic treatment; SD – standard deviation; IQR – interquartile range; IV – intravenous.

In the total cohort of patients with a sinus tract, gram-positive cocci were cultured most often (approximately 70 %), whereas gram-negative rods were cultured less frequently (24 %). In 19 of 72 cases (26.4 %) more than one micro-organism was isolated. In 13 of 72 (18.1 %) cases, the causative micro-organism was not known. For 11 of these patients, no diagnostic procedures were performed to detect the causative micro-organism. For the other two patients, cultures were negative despite the absence of antibiotic treatment at the time of culturing (one sinus tract swab and one tissue biopsy).

The antibiotic therapy and adverse events for those patients who received SAT are summarised in the Supplement (Tables S1 and S2). Sulfamethoxazole–trimethoprim and fluoroquinolones were prescribed most often (in 25 % and 17 % of cases, respectively).

### 3.2 Clinical outcome 

**Figure 1:**
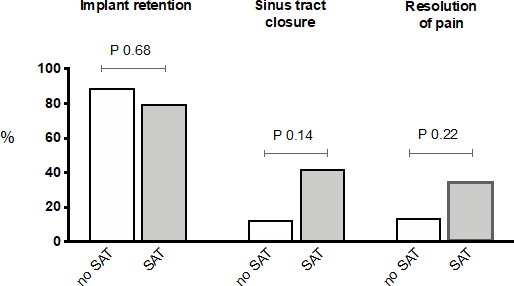
Clinical outcome of patients with and without SAT (suppressive antibiotic treatment).

Baseline characteristics and causative micro-organisms of patients treated with SAT were compared to those for whom SAT was withheld and are summarised in Table 2. Most of the studied variables did not significantly differ between both groups, but SAT was prescribed more often for those patients with a CRP above 50 mg/L (46 % vs. 0 %; p = 0.02). In the patient group for which SAT was not prescribed, the causative micro-organism was less often identified (14 % vs. 44 %; p = 0.05), which is explained by the fact that additional diagnostics were less often performed in this group. Table 3 and Fig. 1 show the primary and secondary end points of the study.

**Table 2:** Patient characteristics and causative micro-organisms for suppressive antibiotic treatment (SAT) vs. no SAT. SAT was stopped before bacteremia developed.

	SAT (n=63)	No SAT (n=9)	p value
Baseline characteristics			
Mean age (SD)	74 (16)	70 (13)	0.10
Male/female	26/37 (41 %/59 %)	2/7 (22 %/78 %)	0.47
Mean BMI (SD)	29.8 (8.4)	25.6 (5.6)	0.12
Smoker	3 (4.8 %)	1 (11.1 %)	0.41
Diabetes mellitus	14 (22.2 %)	1 (11.1 %)	0.67
Chronic kidney disease	3 (4.8 %)	0 (0 %)	1.00
Liver cirrhosis	1 (1.6 %)	1 (11.1 %)	0.24
Joint – Hip – Knee – Shoulder – Elbow	31 (49.2 %) 29 (46.0 %) 2 (3.2 %) 1 (1.6 %)	5 (55.6 %) 3 (33.3 %) 0 (0 %) 1 (11.1 %)	0.36
CRP > 50 mg/L	25/55 (46 %)	0/7 (0 %)	0.021
Haemoglobin > 6 mmol/L	5/43 (11.6 %)	0/5 (0 %)	0.42
Gram positive			
*Staphylococcus aureus*	17 (26.9 %)	1 (11.1 %)	0.52
*Staphylococcus epidermidis*	19 (30.2 %)	2 (22.2 %)	1.00
Coagulase-negative staphylococcus not specified	2 (3.2 %)	0 (0 %)	1.00
*Staphylococcus lugdunensis*	3 (4.8 %)	0 (0 %)	1.00
Methicillin-resistant *Staphylococcus aureus*	3 (4.8 %)	0 (0 %)	1.00
Corynebacterium species	3 (4.8 %)	0 (0 %)	1.00
Gram negative			
*Pseudomonas aeruginosa*	5 (7.9 %)	0 (0 %)	1.00
*Klebsiella pneumoniae*	5 (7.9 %)	0 (0 %)	1.00
*Escherichia coli*	3 (4.8 %)	1 (11.1 %)	0.33
*Proteus mirabilis*	3 (4.8 %)	0 (0 %)	1.00
Other	5 (7.9 %)	1 (11.1 %)	1.0
No micro-organism identified	9 (14.3 %)	4 (44.4 %)	0.05
Polymicrobial	18/63 (28.6 %)	1 (11.1 %)	0.43

**Table 3:** Primary and secondary end points of suppressive antibiotic treatment (SAT) vs. no SAT.

	SAT (n=63)	No SAT (n=9)	p value
**Primary end point**			
Prosthesis retention	79.4 %	88.9 %	0.68
**Secondary end points**			
Prosthetic loosening in initially fixed implants	42 %	0 %	0.08
Need for surgical debridement	6.3 %	0 %	0.44
Sinus tract closure at last follow-up	42.1 %	12.5 %	0.14
Resolution of pain	35.2 %	14.3 %	0.22
Bacteremia with same micro-organism as in PJI	3.2 %	0 %	1.00
CRP > 50 mg/L at last follow-up	12.5 %	16.7 %	0.78
CRP (mg/L) – Baseline (range) – Last follow-up (range) Difference	32.0 (12.0–75.0) 11.7 (4.0–37.0) -12.5 (-41.0 to -0.7)	36.5 (24.5–42.0) 23.0 (14.5–23.0) -10.5 (-22.8–10.4)	0.93 0.26
Haemoglobin < 6 mmol/L at last follow-up	4.7 %	20 %	0.18
Haemoglobin (mmol/L) – Baseline – Last follow-up Difference	7.1 (6.6–8.1) 7.3 (6.6–8.1) -0.1 (-0.6–0.4)	6.83 (6.5–7.2) 6.95 (6.3–7.5) 0.06 (-0.2–0.3)	0.90 0.94
Side effects of SAT	27 %		

Regarding the primary end point, the implant could be retained during follow-up to the same extent in patients receiving SAT vs. those where the SAT was withheld (79.4 % vs. 88.9 %; p = 0.68). For the 14 patients where the implant could not be retained, infections were caused by *Staphylococcus epidermidis* (n = 4), *Staphylococcus aureus* (n = 3), *Staphylococcus lugdunensis* (n = 1), *Staphylococcus saprophyticus* (n = 1), *Escherichia coli* (n = 1), *Corynebacterium striatum* (n = 1), and *Klebsiella pneumoniae* (n = 1). A total of two cases were culture negative. We additionally analysed, in a univariate model, which variables were associated with implant retention during follow-up. The implant could be retained in 88.6 % of patients without prosthetic loosening at baseline, compared to 65.0 % in patients with prosthetic loosening at baseline (p = 0.038). CRP and haemoglobin at baseline, type of joint, the use of SAT, sex, age, BMI, sinus tract closure at last follow-up, bacteremia, type of micro-organism, time between development of the sinus tract and SAT, pain at baseline, and IV antibiotic therapy prior to SAT were not predictive for prosthesis retention during follow-up (data not shown).

Regarding the secondary end points, of the 24 patients with a fixed implant at baseline, 8 developed prosthetic loosening during follow-up. SAT was prescribed in all of these patients (for two of these patients, SAT was started 20 and 44 months after the development of a sinus tract, whereas it was prescribed within 8 months after the development of the sinus tract in the other cases). Surgical debridement during follow-up to control the infection was needed in four patients. All of these four patients were treated with SAT. The causative micro-organisms in these four cases were *S. epidermidis* (n = 2), *S. aureus* (n = 1), and *Klebsiella pneumoniae* (n = 1).

The sinus tract closed in 38.5 % of the total cohort and tended to close more often in the SAT group vs. the no SAT group (42 % vs. 13 %; p = 0.14). In patients receiving SAT, pretreatment with IV antibiotics resulted in a higher rate of sinus tract closure compared with an immediate start of oral SAT, but this difference was not statistically significant (54 % vs. 34 %; p = 0.14). Resolution of pain tended to occur more often in patients receiving SAT compared to those for whom it was withheld (35 % vs. 14 %; p = 0.22), and it was independent of the presence of prosthetic loosening at baseline. No clear differences were observed in the resolution of inflammation and/or anaemia and the occurrence of bacteremia during follow-up between both groups (Table 3). In total, two episodes of bacteremia occurred in the group of patients who received SAT with the same micro-organism as the one causing the PJI (3 %), where one was caused by *S. aureus* and one by *C. perfringens*. No bacteremic episodes were observed in the group of patients where SAT was withheld. During all episodes of SAT, 27 % of patients experienced side effects, of which gastrointestinal manifestations were observed most frequently (Tables S1 and S2).

## 4 Discussion

In this study, we described the outcome of a cohort of patients with a PJI and a draining sinus treated conservatively without an initial surgical intervention. Our study shows that, in most patients (approximately 80 %), the implant could be retained during a follow-up period of at least 2 years, regardless of whether SAT was initiated. SAT was neither able to prevent prosthetic loosening in patients who had a fixed prosthesis at baseline, nor could SAT fully prevent the need for surgical debridement to control the infection during follow-up nor the occurrence of bacteremia. In addition, 27 % of patients experienced side effects during therapy. However, there was a trend towards a beneficial effect of SAT in sinus tract closure and the resolution of pain.

In general, the success rates of PJI patients treated with SAT vary from 23 % to 86 %, but the definition of treatment success that is applied differs between studies. For the vast majority of patients included in our study who received SAT, the implant could be retained during follow-up (Siqueira et al., 2015; Escudero-Sanchez et al., 2020). In a recent multicentre retrospective cohort study performed by Escudero-Sanchez et al. (2020) the implant could be retained in 52.2 % of cases receiving SAT, which is considerably lower compared to our study. This finding can probably be explained by the shorter follow-up period in our study compared to the study of Escudero-Sanchez et al. (2020). The observed difference could not be attributed to the presence of a sinus tract; 133 of 302 (44 %) of the patients in the study of Escudero-Sanchez et al. (2020) had a draining sinus, and its presence was not a predictor for treatment success in the multivariate analysis. Our observation that patients not receiving SAT had a similar primary end point (prosthesis retention), compared to those who received SAT, could not be explained by the type of micro-organism involved or prosthetic loosening at baseline, as these factors were similar between both groups. Only prosthetic loosening at baseline was a predictor of prosthesis extraction during follow-up in logistic regression, but loosening of the prosthesis could neither be prevented with the use of SAT, nor did the prescription of SAT prevent the need for surgical debridement during follow-up to control the infection or the occurrence of bacteremia. These factors are important to take into consideration when considering its use, as the latter reason in particular is sometimes used as an argument to consider using SAT. Regarding other secondary end points, there was a trend towards a beneficial effect of SAT in sinus tract closure (42 % vs. 13 %) and resolution of pain (35 % vs. 14 %), but there was no difference regarding the level of inflammation. In addition, side effects of SAT were described in around 30 % of patients. For this reason, the prescription of SAT should probably be individually tailored, and pain and discomfort of sinus drainage should be taken into account. An alternative approach for prescribing SAT to avoid systemic side effects is the use of subcutaneous SAT (Pouderoux et al., 2019), the local application of bacteriophages (Patey et al., 2018; Tkhilaishivili et al., 2019), or phage lysins (Fischetti, 2018; Schuch et al., 2017; Fowler et al., 2019). These concepts show potential as alternative conservative treatment options (Ferry et al., 2020).

Our study has several strengths and limitations. To our knowledge, this is the first published study to address the use of SAT for PJIs with a draining sinus managed conservatively, and no previous data are available on the clinical outcome of these patients for whom SAT is withheld. In addition, the secondary end points described in our study have not been evaluated in previous literature and are of great value for physicians who are involved in the treatment of these patients. One of the main limitations of our study is the low number of patients not receiving SAT. For this reason, we were unable to fully address the second aim of our study due to the low number of patients included without SAT, and the statistical analysis comparing both groups of patients (SAT vs. no SAT) should be interpreted with caution. Although a large percentage of physicians indicated that withholding SAT in this patient category is realistic and indeed practised by many (Lensen et al., 2020), most patients could not be retrospectively identified and were lost to follow-up. Therefore, prospective trials are needed to have a clear view what happens to these patients in the long term. In addition, patients for whom SAT is withheld probably have a less severe infection. Indeed, in our study, SAT was prescribed more often if patients had a high serum CRP level, rendering it challenging to compare both groups due to bias by indication. Finally, due to the retrospective study design, there was a wide range between the appearance of the sinus tract and the initiation of SAT (i.e. 0–73 months), which may have distorted the interpretation of results (e.g. patients with a greater delay in initiation of SAT may have worse outcomes).

In conclusion, our data suggest that, in PJI patients with a draining sinus, SAT should only be considered in a subset of patients. SAT may reduce pain and favour closure of the sinus tract in certain individuals, but the prescription of SAT does not seem to have any influence on the prevention of prosthetic loosening and other infectious complications. Larger randomised trials are needed to prospectively compare SAT vs. no SAT in patients with draining sinus.

## Supplement

10.5194/jbji-6-313-2021-supplement The supplement related to this article is available online at: https://doi.org/10.5194/jbji-6-313-2021-supplement.

## Data Availability

No ethical approval has been obtained to share the analysed data online.

## Team list

Other members of the ESGIAI who participated in the study are Samo Roškar, Julia Praena, Taiana Cunha Ribeiro, Francesco Taccari, Anne Méheut, Yvonne Achermann, Octavian Andronic, Victor Asensi, Claudia Moran-Castaño, and Alfredo Jover-Sáenz.

## References

[bib1.bib1] Escudero-Sanchez R, Senneville E, Digumber M, Soriano A, Del Toro MD, Bahamonde A, Del Pozo JL, Guio L, Murillo O, Rico A, García-País MJ, Rodríguez-Pardo D, Iribarren JA, Fernández M, Benito N, Fresco G, Muriel A, Ariza J, Cobo J (2020). Suppressive antibiotic therapy in prosthetic joint infections: amulticentre cohort study. Clin Microbiol Infect.

[bib1.bib2] Ferry T, Batailler C, Brosset S, Kolenda C, Goutelle S, Sappey-Marinier E, Josse J, Laurent F, Lustig S, Lyon BJI Study Group (2020). Medical innovations to maintain the function in patients with chronic PJI for
whom explantation is not desirable: a pathophysiology-, multidisciplinary-, and experience-based approach. SICOT-J.

[bib1.bib3] Fischetti VA (2018). Development of phage lysins as novel therapeutics: A historical perspective. Viruses.

[bib1.bib4] Fowler V G Jr, Das A F, Lipka-Diamond J, Schuch R, Pomerantz R, Jáuregui-Peredo L, Bressler A, Evans D, Moran GJ, Rupp ME, Wise R, Corey GR, Zervos M, Douglas PS, Cassino C (2019). Exebacase (Lysin CF-301) improved clinical responder rates in methicillin
resistant Staphylococcus aureus bacteremia including endocarditis compared to standard of care
antibiotics alone in a first-in patient phase 2 study.

[bib1.bib5] Lensen K-J, Escudero-Sanchez R, Cobo J, Soriano A, Wouthuyzen-Bakker M (2020). Chronic prosthetic joint infections with a draining sinus. Who should receive suppressive antibiotic treatment?. J Bone Joint Infect.

[bib1.bib6] Osmon DR, Berbari EF, Berendt AR, Lew D, Zimmerli W, Steckelberg JM, Rao N, Hanssen A, Wilson WR, Infectious Diseases Society of America (2013). Diagnosis and management of prosthetic joint infection: clinical practice guidelines
by the infectious diseases society of America. Clin Infect Dis.

[bib1.bib7] Patey O, McCallin S, Mazure H, Liddle M, Smithyman A, Dublanchet A (2018). Clinical indications and compassionate use of phage therapy: Personal experience and literature review with a focus on osteoarticular infections. Viruses.

[bib1.bib8] Pouderoux C, Becker A, Goutelle S, Lustig S, Triffault-Fillit C, Daoud F, Fessy MH, Cohen S, Laurent F, Chidiac C, Valour F, Ferry T, Lyon Bone Joint Infection Study Group (2019). Subcutaneous suppressive antibiotic therapy for bone and joint infections:
Safety and outcome in a cohort of 10 patients. J Antimicrob Chemother.

[bib1.bib9] PPradier M, Robineau O, Boucher A, Titecat M, Blondiaux N, Valette M, Loïez C, Beltrand E, Nguyen S, Dézeque H, Migaud H, Senneville E (2018). Suppressive antibiotic therapy with oral tetracyclines for prothestic joint infections: a retrospective study of 78 patients. Infection.

[bib1.bib10] Prendki V, Zeller V, Passeron D, Desplaces N, Mamoudy P, Stirnemann J, Marmor S, Ziza JM (2014). Outcome of patients over 80 years of age on prolonged suppressive antibiotic therapy for at least 6 months for prosthetic joint infection. Int J Infect Dis.

[bib1.bib11] Rao N, Crossett LS, Sinha RK, Le Frock JL (2003). Long-term suppression of infection in total joint arthroplasty. Clin Orthop Relat Res.

[bib1.bib12] Schuch R, Khan BK, Raz A, Rotolo JA, Wittekind M (2017). Bacteriophage lysin CF-301, a potent antistaphylococcal biofilm agent. Antimicrob Agents Chemother.

[bib1.bib13] Segreti J, Nelson JA, Trenholme GM (1998). Prolonged suppressive antibiotic therapy for infected orthopedic prostheses. Clin Infect Dis.

[bib1.bib14] Siqueira MB, Saleh A, Klika AK, O'Rourke C, Schmitt S, Higuera CA, Barsoum WK (2015). Chronic suppression of periprosthetic joint infections with oral antibiotics increases infection-free survivorship. J Bone Jt Surg Am.

[bib1.bib15] Tkhilaishvili T, Winkler T, Müller M, Perka C, Trampuz A (2019). Bacteriophages as adjuvant to antibiotics for the treatment of periprosthetic joint infection caused by multidrug-resistant Pseudomonas aeruginosa. Antimicrob Agents Chemother.

[bib1.bib16] Tsukayama DT, Wicklund B, Gustilo RB (1991). Suppressive antibiotic therapy in chronic prosthetic joint infections. Orthopedics.

[bib1.bib17] Wouthuyzen-Bakker M, Nijman JM, Kampinga GA, van Assen S, Jutte PC (2017). Efficacy of antibiotic suppressive therapy in patients with a prosthetic joint infection. J Bone Joint Infect.

