# Supplementary material for: The efficacy of suppressive antibiotic treatment in patients managed non-operatively for periprosthetic joint infection and a draining sinus
Source: J Bone Jt Infect. 2021 Aug 17;6(7):313–9. doi: 10.5194/jbji-6-313-2021 (PMC8375509; doi:10.5194/jbji-6-313-2021)
Supplement: The supplement related to this article is available online at: https://doi.org/10.5194/jbji-6-313-2021-supplement. [file jbji-6-313-supplement.pdf]

Supplement of J. Bone Joint Infect., 6, 313–319, 2021
https://doi.org/10.5194/jbji-6-313-2021-supplement
© Author(s) 2021. CC BY 4.0 License.

Supplement of

The efficacy of suppressive antibiotic treatment in patients managed non-operatively for periprosthetic joint infection and a draining sinus

Karel-Jan Dag François Lensen et al.

Correspondence to: Marjan Wouthuyzen-Bakker (m.wouthuyzen-bakker@umcg.nl)

The copyright of individual parts of the supplement might differ from the article licence.

Antibiotic	First SAT	Second SAT	Third SAT
Sulfamethoxazol-trimethoprim	16/63 (25.4%)	16/38 (42.1%)	4/18 (22.2%)
Amoxicillin	5/63 (7.9%)	0/38 (0%)	0/18 (0%)
Amoxicillin-clavulanic acid	3/63 (4.7%)	3/38 (7.9%)	1/18 (5.6%)
Ciprofloxacin	7/63 (11.1%)	3/38 (7.9%)	0/18 (0%)
Levofloxacin	4/63 (6.3%)	0/38 (0%)	1/18 (5.6%)
Doxycyclin	6/63 (9.5%)	0/38 (0%)	2/18 (11.1%)
Clindamycin	4/63 (6.3%)	4/38 (10.5%)	2/18 (11.1%)
Minocyclin	4/63 (6.3%)	1/38 (2.6%)	0/18 (0%)
Cefadrox	2/63 (3.2%)	1/38 (2.6%)	1/18 (5.6%)
Other*	12/63 (19.3%)	10/38 (16.3%)	7/18 (38.9%)

Supplemental Table 1. The prescription of suppressive antibiotic treatment (SAT). The Table indicates different regimens used per individual (First, second and third period).

Adverse event	First SAT	Second SAT	Third SAT
Total number	17/63 (27.0%)	8/37 (21.6%)	4/18 (22.2%)
Specified			
Gastro-intestinal	10/63 (15.9%)	1/37 (2.6%)	1/18 (5.6%)
Hematological	2/63 (3.2%)	2/37 (5.3%)	2/18 (11.1%)
Musculoskeletal	2/63 (3.2%)	0/37 (0%)	0/18 (0%)
Dermatological	2/63 (3.2%)	0/37 (0%)	0/18 (0%)
Allergy	0/63 (0%)	4/37 (10.5%)	0/18 (0%)
Other	1/63 (1.6%)	1/37 (2.6%)	1/18 (5.6%)

Supplemental Table 2. Adverse events in PJI patients with a draining sinus on suppressive antibiotic treatment (SAT). The Table indicates different regimens used per individual (First, second and third period).
